# Clinical impact and risk prediction of pulmonary barotrauma in non-HIV *Pneumocystis jirovecii* pneumonia: a 15-year single-center retrospective study

**DOI:** 10.3389/fmed.2026.1761288

**Published:** 2026-04-28

**Authors:** Bangchao Zhao, Xi Wang, Zhou Jin, Guangfa Wang, Yuxiao Wu, Kunyao Yu, Bowen Zhang, Jing Ma

**Affiliations:** 1 Department of Respiratory Medicine and Critical Care Medicine, Peking University First Hospital, Beijing, China; 2 Institute of Medical Technology, Peking University Health Science Center, Beijing, China

**Keywords:** non HIV patient, oxygenation index, *Pneumocystis jirovecii* pneumonia, pulmonary barotrauma, risk prediction model

## Abstract

**Background:**

Pulmonary barotrauma is a severe complication of *Pneumocystis jirovecii* pneumonia (PJP), yet its prognostic significance in non-human immunodeficiency virus (HIV) infection populations remains uncharacterized. The aim of this study was to analyze the incidence and associated risk factors of pulmonary barotrauma in non-HIV patients with PJP.

**Methods:**

We retrospectively reviewed all non-HIV PJP patients admitted to our hospital from January 2009 to December 2024. Patients were divided into two groups based on whether they developed barotrauma. Through multivariate binary regression analysis, independent risk factors for the occurrence of barotrauma were identified and a predictive model was developed.

**Results:**

Among 334 patients, 40 (12.0%) developed barotrauma: pneumothorax (*n* = 23) and isolated subcutaneous emphysema or pneumomediastinum (*n* = 17). Barotrauma patients had higher rates of intensive care unit admission (85.0% vs. 37.4%), invasive mechanical ventilation (75.0% vs. 20.7%), and mortality (72.5% vs. 23.8%). They also had a higher proportion of patients with a smoking history (50.0% vs. 33.7%). Admission labs showed elevated white blood cell count (8.7 vs. 7.4 × 10^9^/L), lactate dehydrogenase (506 vs. 383 IU/L), C-reactive protein (63.68 vs. 50.12 mg/L), 1,3-β-D-glucan (312.98 vs. 156.32 pg./mL), and bronchoalveolar lavage fluid neutrophils (53.0% vs. 20.5%), along with reduced albumin (28.3 vs. 30.6 g/L) and oxygenation index (PaO_2_/FiO_2_ ratio) (165.52 vs. 266.67 mmHg). Multivariable binary logistic regression identified PaO_2_/FiO_2_ as an independent predictor of barotrauma (OR = 0.993, 95% CI 0.989–0.998, *p* = 0.003). The final multivariable model demonstrated good discriminative performance, yielding an area under the curve of 0.751 (95% CI 0.674–0.828).

**Conclusion:**

In non-HIV PJP, pulmonary barotrauma is not only common but also strongly associated with critical illness and mortality. Impaired oxygenation at admission independently predicts its occurrence. This finding provides a clinically applicable model that may enable earlier risk stratification and guide closer monitoring and management in high-risk patients.

## 1 Introduction

Pulmonary barotrauma refers to the extravasation of gas beyond the alveoli into the pulmonary interstitium or body cavities, resulting in extra-alveolar air accumulation, such as pneumothorax, pneumomediastinum, and subcutaneous emphysema (1). Pathophysiologically, it is caused by alveolar rupture with subsequent multidirectional air dissection along the lung interstitium. In patients with acute respiratory distress syndrome (ARDS), the incidence of pulmonary barotrauma is estimated to be approximately 5–10%, and even higher in coronavirus disease 2019 (COVID-19)-associated ARDS (2, 3). Pulmonary barotrauma is frequently accompanied by severe disease progression and high mortality, prolonging hospital stay and imposing a substantial healthcare burden.

Several studies have identified barotrauma as a risk factor for mortality in *Pneumocystis jirovecii* pneumonia (PJP) (4–6). However, research on the risk factors for barotrauma in non-human immunodeficiency virus (HIV)-infected PJP patients is currently limited, with only one small-scale (*n* = 119), single-center retrospective study published in 2022 (7). Identifying the risk factors for barotrauma in non-HIV-infected PJP patients is crucial for early identification of high-risk populations, timely intervention, and improving patient outcomes.

## 2 Materials and methods

### 2.1 Study design and participants

**Figure 1:**
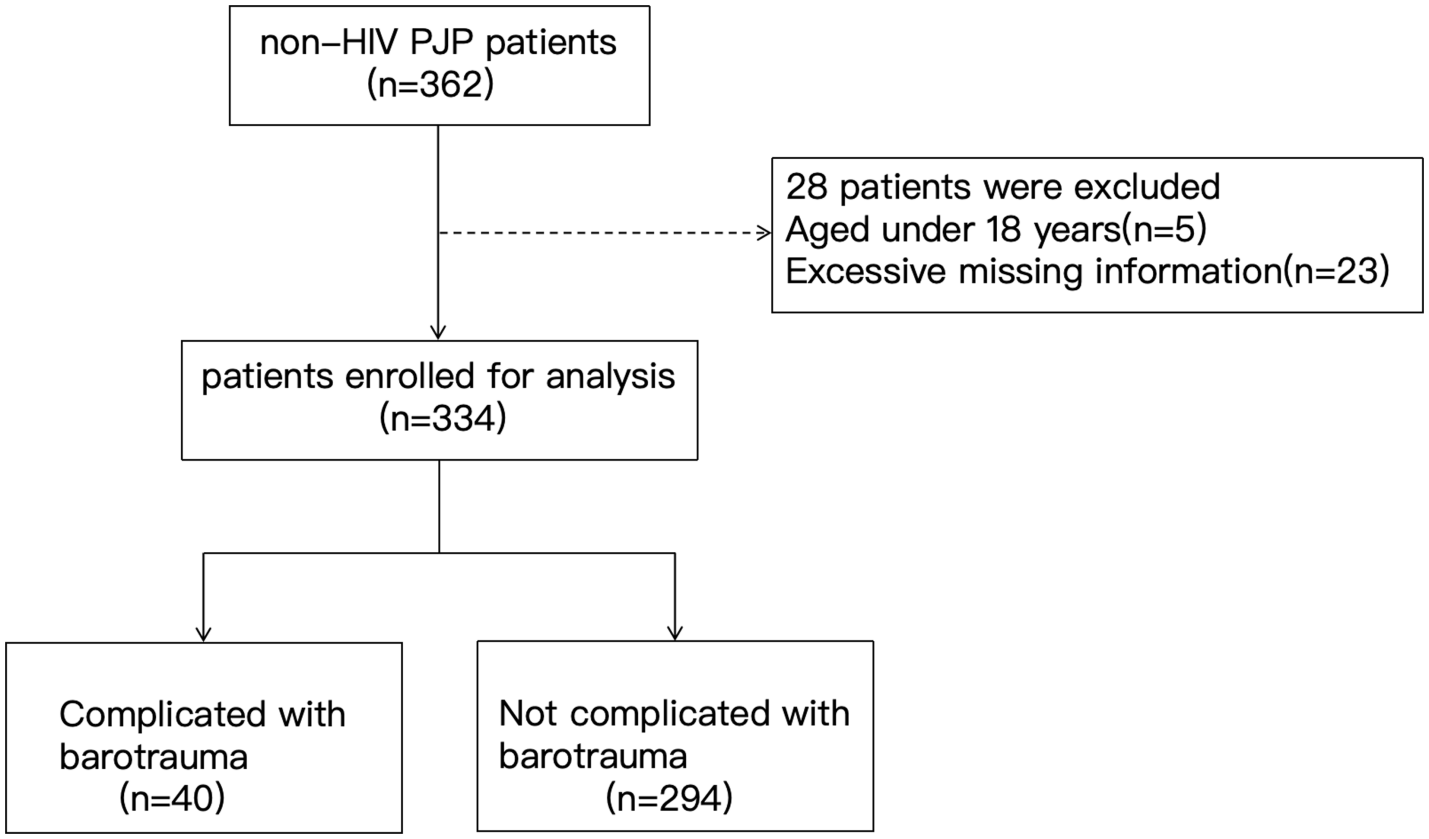
Flowchart of patient selection. PJP, *Pneumocystis jirovecii* pneumonia; HIV, human immunodeficiency virus.

This study is a retrospective cohort study, enrolling all non-HIV-infected patients with PJP hospitalized in the Peking University First Hospital from January 2009 to December 2024 (*n* = 334) (Figure 1). The study was approved by the Ethics Committee of our hospital (Ethics Approval No: 2025R0208-0001). Due to the retrospective data analysis, written informed consent was waived.

### 2.2 Diagnosis of PJP cases

Patients were diagnosed with PJP if they met the following criteria: (1) Host factor: immunocompromise (e.g., hematologic malignancy, solid organ transplant, prolonged corticosteroid use with a dose equivalent to ≥20 mg of prednisone daily for ≥3 weeks, or the use of a second immunosuppressive drug). (2) Clinical-radiologic criteria: symptoms including fever, nonproductive cough, and progressive dyspnea. (3) Imaging: bilateral diffuse ground-glass opacities or interstitial infiltrates on chest computed tomography. (4) Direct mycologic evidence: detection of *P. jirovecii* in tissue or respiratory specimens (e.g., bronchoalveolar lavage fluid (BALF) or induced sputum) using Gomori methenamine silver staining or molecular diagnostic methods. Quantitative real-time polymerase chain reaction was considered supportive of active infection when low cycle-threshold values (≤34–35) were observed. Next-generation sequencing (NGS) was regarded as positive when *P. jirovecii* reads exceeded the laboratory-defined threshold for pathogen detection or demonstrated a distinctly higher relative abundance compared with background microbial taxa. As NGS cutoffs vary across platforms and analytic pipelines, interpretation was based on institution-specific validation criteria and clinical–radiological correlation (8).

### 2.3 Definition of barotrauma and grouping

The primary outcome was pulmonary barotrauma, defined as clinically or radiologically confirmed pneumothorax, pneumomediastinum, or subcutaneous emphysema. The non-HIV PJP patients were divided into two groups based on whether they developed barotrauma during hospitalization. All images were evaluated by two independent physicians, and disputes were adjudicated by a third expert. The time of barotrauma occurrence was recorded as the date of first diagnosis.

### 2.4 Variable collection and data verification

Clinical parameters extracted from electronic medical records included demographic characteristics (age, sex), comorbidities, smoking history, immunosuppressive therapy, admission laboratory indicators, BALF analysis, respiratory support, treatment details, barotrauma occurrence, intensive care unit (ICU)-related information (admission, length of stay), and prognosis. All data were independently entered by two researchers, and any discrepancies were reconciled by referring to the original medical records.

### 2.5 Statistical analysis

For continuous variables, normally distributed variables are reported as mean and standard deviation, while skewed variables are reported as median and interquartile range (IQR). Categorical variables are expressed as frequencies and percentages. Comparisons of continuous variables were performed using t-tests or Mann–Whitney *U* tests. Comparisons of categorical variables were performed using chi-square tests or Fisher’s exact tests. Differences in continuous variables over time were compared using repeated measures analysis of variance. Logistic regression models were used to identify risk factors for poor outcomes. Variables with *p* < 0.05 in univariate analyses were screened, after which clinically meaningful and non-collinear predictors were selected to construct the final multivariable logistic regression model to avoid overfitting. All statistical analyses were performed using the SPSS software version 27.0 (IBM, Armonk, NY, United States). *p* < 0.05 was considered statistically significant, and all tests were 2-tailed.

To ensure that the multivariable model reflected true predictive value rather than concurrent associations, only the earliest available clinical and laboratory parameters obtained at admission were considered for analysis. All variables with statistically significant differences in univariate comparisons, along with clinically relevant covariates, were evaluated as candidate predictors. From these candidates, a subset of variables was selected for inclusion in the final multivariable logistic regression model based on statistical performance and clinical interpretability, with the aim of constructing a parsimonious model suitable for early risk prediction.

The 1,3-β-D-glucan (*G* test) was measured using two different analytical methods (turbidimetric and colorimetric assays) following a change in the laboratory protocol in 2022. To account for potential inter-assay variability, *G* test values were standardized within each method using z-scores following data preprocessing. Approximately 10% of *G* test values were missing. These missing values were imputed using the median value within each assay group (implemented using SPSS software). (As a sensitivity analysis, multiple imputation by chained equations was also performed, yielding similar results). Those standardized values were subsequently entered into the multivariable logistic regression model.

## 3 Results

### 3.1 Characteristics of enrolled patients

A total of 334 non-HIV PJP patients were enrolled in this study, of whom 221 were male (66.2%), with a median age of 57 years (IQR 42.8–66.0). The diagnosis was confirmed by microscopic identification of cysts in 248 patients (74.3%). Patients were categorized into the barotrauma group (B-PJP, *n* = 40) and the non-barotrauma group (NB-PJP, *n* = 294). Baseline demographic and clinical characteristics of the two groups are summarized in Table 1. The groups were well-balanced in terms of sex, age, and body mass index (BMI), with no statistically significant differences observed. A significantly higher proportion of smokers was observed in the B-PJP group (50.0%) compared to the NB-PJP group (33.7%; *p* = 0.043). The prevalence of a history of hematopoietic stem cell transplantation was significantly lower in the B-PJP group (0%) than in the NB-PJP group (11.9%; *p* = 0.013). Conversely, chronic kidney disease (CKD) was significantly more prevalent in the B-PJP group (37.5%) than in the NB-PJP group (20.7%; *p* = 0.018).

**Table 1:** Baseline characteristics of non-HIV PJP patients with and without pulmonary barotrauma.

Characteristics	NB-PJP	B-PJP	*P*
	(*n* = 294)	(*n* = 40)	
Sex, male	193(65.7)	28(70.0)	0.585
*Age, years	57(43,66)	61(46,69)	0.647
*BMI, kg/m^2^	22.72(20.08,25.39)	23.61(19.88,25.65)	0.715
Smoking history	99(33.7)	20(50.0)	0.043
Underlying diseases			
Solid organ transplantation	19(6.5)	1(2.5)	0.488
HSCT	35(12)	0(0)	0.013
Hematological malignancies	33(11.2)	3(7.5)	0.596
Solid tumor	33(11.2)	6(15.0)	0.440
Autoimmune diseases	78(26.5)	7(17.5)	0.219
Chronic kidney disease	61(20.8)	15(37.5)	0.018
ABD	17(5.8)	3(7.5)	0.719
Others	18(6.1)	5(12.5)	0.136
Comorbidities			
Hypertension	138(47)	16(40)	0.409
Diabetes	73(24.8)	11(27.5)	0.715
Coronary heart disease	26(8.8)	2(5.0)	0.411
Interstitial lung disease	29(9.9)	4(10.0)	1.000
Initial symptoms			
Fever	206(70.1)	27(67.5)	0.740
Cough	21(7.1)	2(5.0)	0.613
Dyspnea	67(22.8)	11(27.5)	0.506

Variables marked with asterisks (*) represent continuous data, which are presented as the median (interquartile range, IQR). Others are categorical variables, presented as *n* (%). BMI, body mass index; HSCT, hematopoietic stem cell transplantation; ABD, autoimmune blistering diseases.

### 3.2 Incidence of barotrauma and clinical correlations

Table 2 summarizes the subtype-specific clinical features, detection, and management of barotrauma in patients with PJP. Pulmonary barotrauma occurred in 40 patients (12.0%), presenting as pneumothorax (*n* = 23) or isolated subcutaneous/mediastinal emphysema (*n* = 17). Among those with pneumothorax, 14 cases were complicated by concurrent subcutaneous or mediastinal emphysema. The median time from PJP onset, defined as the initial presentation of PJP-related symptoms, to barotrauma was 18.5 days (IQR, 5–43 days). Most patients developed pneumothorax on the right side or bilaterally. New-onset clinical symptoms and signs represented the predominant means of identifying pneumothorax in this cohort, whereas other types of barotrauma were identified primarily by imaging examinations.

**Table 2:** Subtype-specific presentation, detection, and management of barotrauma in patients with B-PJP.

Characteristics, *n* (%)	Pneumothorax (*n* = 23)	Other barotrauma* (*n* = 17)
Laterality of pneumothorax		
Left-sided	5(21.7)	–
Right-sided	9(39.1)	–
Bilateral	9(39.1)	–
Recognition		
Promptly recognized due to new clinical manifestations^†^	18(78.3)	1(5.9)
Detected on physical examination	4(17.4)	3(17.6)
Detected radiographically (CT or chest X-ray)	1(4.3)	13(76.5)
Respiratory support at barotrauma diagnosis		
IMV	9(39.1)	6(35.3)
NIV	13(56.6)	8(47.1)
Conventional oxygen therapy	1(4.3)	3(17.6)
Management		
Conservative management only	10(43.5)	17(100)
Needle aspiration	1(4.3)	–
Chest tube drainage	12(52.2)	–

*Other barotrauma included subcutaneous emphysema and/or pneumomediastinum.

^†^New clinical manifestations included sudden chest pain, dyspnea or acute worsening of oxygenation. IMV, invasive mechanical ventilation; NIV, non-invasive ventilation; CT, computed tomography.

In the B-PJP cohort, 21 patients (52.5%) developed barotrauma during non-invasive ventilation (NIV), and 15 patients (37.5%) experienced barotrauma during invasive mechanical ventilation (IMV). In addition, four cases (10%) of barotrauma occurred during conventional oxygen therapy.

The median interval from IMV initiation to barotrauma diagnosis was 192 h (IQR 72–552). Notably, 33.3% (5/15) of cases occurred within 72 h of IMV initiation. In the patients who developed barotrauma during NIV, the median interval from NIV initiation to diagnosis was 72 h (IQR 30–156). Of these, 23.8% (5/21) developed it within 24 h.

In this cohort, the management of pneumothorax followed guidelines for pleural diseases (9) and standard clinical practice, including conservative management, needle aspiration, and chest tube drainage. Among the 23 patients with pneumothorax, one underwent needle aspiration, 12 received tube thoracostomy, and the remaining 10 were managed conservatively with ventilator adjustment, supplemental oxygen, and close observation. No moderate-to-severe procedure-related complications were observed in the management of pneumothorax. The other 17 patients, who presented with other forms of barotrauma, received non-invasively conservative treatment only.

Among the 23 pneumothorax cases, 17 patients died, resulting in a mortality rate of 73.9% (17/23). Of the remaining 17 patients with other forms of barotrauma, 12 patients died, corresponding to a mortality rate of 70.5% (12/17). Comparison of mortality between the two types of barotrauma showed no statistically significant difference (73.9% vs. 70.5%; *p* = 0.83).

The median interval from symptom onset to treatment initiation did not differ between the NB-PJP and B-PJP groups (9.0 [IQR 5.0–17.0] vs. 8.5 [IQR 4.3–15.5] days, *p* = 0.586). As shown in Table 3, patients with B-PJP exhibited substantially higher rates of ICU admission (85.0% vs. 37.4%; *p* < 0.001), NIV (75.0% vs. 30.6%; *p* < 0.001), IMV (75.0% vs. 20.7%; *p* < 0.001), and in-hospital mortality (72.5% vs. 23.8%; *p* < 0.001). The duration of IMV tended to be longer in the B-PJP group (216 vs. 120 h), although this difference did not reach statistical significance (*p* = 0.063). Length of ICU stay and total hospital stay were comparable between groups.

**Table 3:** Comparison of treatment and outcome between NB-PJP and B-PJP.

Characteristics	NB-PJP (*n* = 294)	B-PJP (*n* = 40)	*P*
*Time from PJP onset to treatment initiation (days)	9.0(5.0,17.0)	8.5(4.3,15.5)	0.586
Admission to ICU	110(37.4%)	34(85.0%)	<0.001
*Length of ICU stay (days)	11(5,18)	16(6,25)	0.093
NIV	90(30.6%)	30(75.0%)	<0.001
IMV	61(20.7%)	30(75.0%)	<0.001
*Duration of IMV (hours)	120(76,303)	216(122,464)	0.063
Hospital mortality	70(23.8%)	29(72.5%)	<0.001
*Length of hospital stay (days)	19(11,29)	19.5(10,32)	0.462

Variables marked with asterisks (*) represent continuous data, which are presented as the median (interquartile range, IQR). Others are categorical variables, presented as *n* (%). PJP, *Pneumocystis jirovecii* pneumonia; ICU, intensive care unit; NIV, non-invasive ventilation; IMV, invasive mechanical ventilation.

### 3.3 Laboratory and pathological examination results

Table 4 summarizes significant differences in clinical and laboratory parameters between the B-PJP and NB-PJP groups. The B-PJP group demonstrated markedly higher median levels of lactate dehydrogenase (LDH), C-reactive protein.

**Table 4:** Comparison of laboratory data and pathological examination of patients with and without barotrauma.

Characteristics	NB-PJP (*n* = 294)	B-PJP (*n* = 40)	*P*
Clinical parameters			
WBC count, ×10^9^/L	7.4(4.70, 10.05)	8.7(6.70, 11.95)	0.010
Ly ratio (%)	8.2(4.55, 12.35)	7(4.80, 12.30)	0.643
Ly count, ×10^9^/L	0.5(0.30, 0.90)	0.65(0.40, 0.93)	0.157
ESR, mm/h	52.5(27, 85)	63.5(33, 81)	0.437
ALT, IU/L	22.0(15.0, 41.0)	27.5(14.3, 40.3)	0.614
TP, g/L	58.50(52.00, 64.00)	57.35(47.35, 63.95)	0.206
Alb, g/L	30.60(26.70, 34.65)	28.25(24.23, 32.08)	0.018
Scr, μmol/L	93.80(69.65, 162.16)	95.20(72.25, 146.66)	0.800
BUN, mmol/L	7.79(5.46, 14.11)	10.20(7.61, 13.56)	0.051
LDH, IU/L	383.0(293.5, 514.5)	506.0(440.0, 867.7)	<0.001
CRP, mg/L	50.12(20.49, 96.63)	63.68(44.72, 126.80)	0.038
CD4 + T cell, cells/μL	196.00(87.87, 425.57)	195.86(92.22, 288.50)	0.762
CD8 + T cell, cells/μL	234.29(119.26, 442.50)	274.07(136.84, 423.88)	0.743
CD3 + T cell, cells/μL	496.22(233.63, 955.19)	487.13(315.59, 778.84)	0.857
*G* test, pg./mL	156.32(5.00, 353.73)	312.98(33.53, 711.18)	0.001
PCT, ng/mL	0.17(0.07, 0.44)	0.26(0.12, 0.85)	0.070
PaO_2_/FiO_2_, mmHg	266.67(189.63, 328.57)	165.52(117.53, 247.92)	0.001
*Respiratory failure	177(60.2%)	33(82.5%)	0.006
*CMV infection	149(50.68%)	24(60.00%)	0.268
BALF test (*n* = 285)			
Nc density,10^6^/mL	0.15(0.08, 0.23)	0.11(0.04, 0.18)	0.055
Mo, %	37.0% (20.0, 50.8%)	25.0% (14.0, 39.5%)	0.019
Ne, %	20.5% (8.0, 50.5%)	53.0% (23.5, 70.5%)	<0.001
Ly, %	27.5% (10.0, 47.0%)	17.0% (9.0, 24.5%)	0.024
*Presence of cysts	211(71.8%)	37(92.5%)	0.005

Variables marked with an asterisk (*) are categorical variables, presented as *n* (%). The remaining continuous variables are expressed as median (interquartile range, IQR). Respiratory failure is based on the result of the first blood gas analysis, WBC, white blood cell; Ly, lymphocyte; ESR, erythrocyte sedimentation rate; ALT, alanine transaminase; TP, total protein; Alb, albumin; Scr, serum creatinine; BUN, blood urea nitrogen; LDH, lactate dehydrogenase; CRP, C-reactive protein; *G* test, 1,3-β-D-glucan test; PCT, procalcitonin; PaO_2_/FiO_2_, oxygenation index; CMV, cytomegalovirus; BALF, bronchoalveolar lavage fluid; Nc, nucleated cell; Mo, macrophages; Ne, neutrophils.

(CRP), *G* test, and white blood cell (WBC) count, coupled with lower median PaO_2_/FiO_2_ and albumin (Alb) level. In addition, respiratory failure on initial arterial blood gas analysis was more common in the B-PJP group than in the NB-PJP group (82.5% vs. 60.2%, *p* = 0.006). Bronchoalveolar lavage fluid analysis further revealed a higher neutrophil percentage but lower macrophage and lymphocyte percentages, along with a higher detection rate of *P. jirovecii* cysts in the B-PJP group. No significant intergroup differences were noted in T-cell subsets, cytomegalovirus (CMV) co-infection rates, and other clinical parameters.

### 3.4 Prognostic factors by univariate analysis

As shown in Table 5, univariate analysis identified variables significantly associated with pulmonary barotrauma. Risk factors included clinical characteristics such as smoking history, history of CKD, ICU admission, use of IMV or NIV, and positive detection of *P. jirovecii cysts*. Laboratory and physiological parameters positively associated with barotrauma risk included elevated WBC count, LDH, CRP, *G* test, a longer diagnosis-to-treatment interval, and a higher BALF neutrophil percentage. Conversely, higher PaO_2_/FiO_2_ and greater BALF macrophage and lymphocyte percentages emerged as protective factors.

**Table 5:** Univariate logistic regression analysis of factors associated with barotrauma in Non-HIV PJP patients.

Variables	Univariate logistic regression		
	OR	95% CI	*P*
Sex	1.221	0.596–2.503	0.586
Age	1.003	0.983–1.023	0.797
BMI	0.996	0.969–1.024	0.800
Smoking history	1.970	1.013–3.831	0.046
Underlying disease			
Solid organ transplantation	2.695	0.351–20.695	0.341
Hematological malignancies	0.641	0.187–2.196	0.479
Solid tumor	1.396	0.545–3.574	0.487
Autoimmune diseases	0.587	0.250–1.382	0.223
Chronic kidney disease	2.292	1.139–4.613	0.020
ABD	0.757	0.212–2.707	0.668
Others	0.741	0.290–1.894	0.531
Comorbidities			
Hypertension	0.754	0.385–1.477	0.410
Diabetes	0.871	0.414–1.830	0.715
Coronary heart disease	1.843	0.421–8.080	0.417
Interstitial lung disease	0.985	0.327–2.964	0.978
Initial symptoms			
Fever	1.127	0.556–2.286	0.740
Cough	0.729	0.352–1.509	0.395
Dyspnea	1.462	0.330–6.482	0.618
Clinical parameters			
WBC count	1.116	1.025–1.216	0.011
Ly ratio	0.975	0.935–1.016	0.229
Ly count	1.162	0.756–1.787	0.493
ESR	1.003	0.992–1.014	0.586
ALT	0.997	0.989–1.006	0.536
TP	0.978	0.945–1.012	0.206
Alb	0.950	0.902–1.001	0.054
Scr	1.000	0.998–1.002	0.823
BUN	1.006	0.973–1.040	0.735
LDH	1.002	1.001–1.003	0.003
CRP	1.004	1.000–1.009	0.044
*G* test	1.446	1.044–2.002	0.026
PCT	1.019	0.945–1.098	0.625
CMV infection	1.460	0.745–2.860	0.270
PaO_2_/FiO_2_	0.991	0.988–0.995	<0.001
Treatment			
Time from diagnosis to treatment initiation	1.116	1.006–1.239	0.039
ICU admission	9.479	3.856–23.301	<0.001
IMV	10.07	4.761–21.299	<0.001
NIV	5.976	2.860–12.488	<0.001
BALF test (*n* = 285)			
Mo	0.978	0.958–0.998	0.027
Ne	1.022	1.009–1.035	0.001
Ly	0.976	0.958–0.996	0.017
Microscopically positive of *Pneumocystis jirovecii* cysts	4.852	1.456–16.167	0.010

BMI, body mass index; ABD, autoimmune blistering diseases; WBC, white blood cell; Ly, lymphocyte; ESR, erythrocyte sedimentation rate; ALT, alanine transaminase; TP, total protein; Alb, albumin; Scr, serum creatinine; BUN, blood urea nitrogen; LDH, lactate dehydrogenase; CRP, C-reactive protein; *G* test, 1,3-β-D-glucan test; PCT, procalcitonin; CMV, cytomegalovirus; PaO_2_/FiO_2_, oxygenation index; ICU, intensive care unit; NIV, non-invasive ventilation; IMV, invasive mechanical ventilation; BALF, bronchoalveolar lavage fluid; Mo, macrophages; Ne, neutrophils.

### 3.5 Multivariate analysis

Variables for the multivariable logistic regression model were selected *a priori* based on their clinical relevance and univariate associations (*p* < 0.05, as appropriate). Given the limited number of barotrauma events (*n* = 40), we restricted the number of predictors in the multivariable model to eight to maintain an adequate events-per-variable ratio and to reduce the risk of overfitting. Candidate variables with *p* < 0.05 in univariable analyses were considered for inclusion. Variables reflecting subsequent disease severity or treatment escalation (ICU admission, IMV, NIV) were not entered because our aim was to build a baseline risk prediction model at the time of PJP diagnosis. Due to excessive data missing, the BALF test results were not included in the multivariate analysis.

In multivariable regression, WBC remained a significant predictor of barotrauma (*β* = 0.012, *p* = 0.019), whereas CRP did not (*β* ≈ 0, *p* = 0.146). The model *R*^2^ was very low, indicating minimal additional explanatory value contributed by CRP. Therefore, WBC was retained and CRP excluded to avoid redundant inflammatory markers and to ensure a more parsimonious model without loss of predictive information.

Serum Alb (OR 0.95, 95% CI 0.902–1.001, *p* = 0.054) demonstrated borderline significance in univariate analysis; however, based on its established biological relevance, it was included in the multivariable model to avoid omission of an important clinical covariate.

Among the remaining correlated markers of inflammation and disease severity, we retained those with the strongest clinical relevance and independent association with barotrauma. Comorbidities with weaker or non-independent effects (e.g., CKD) were therefore excluded from the final model.

**Figure 2:**
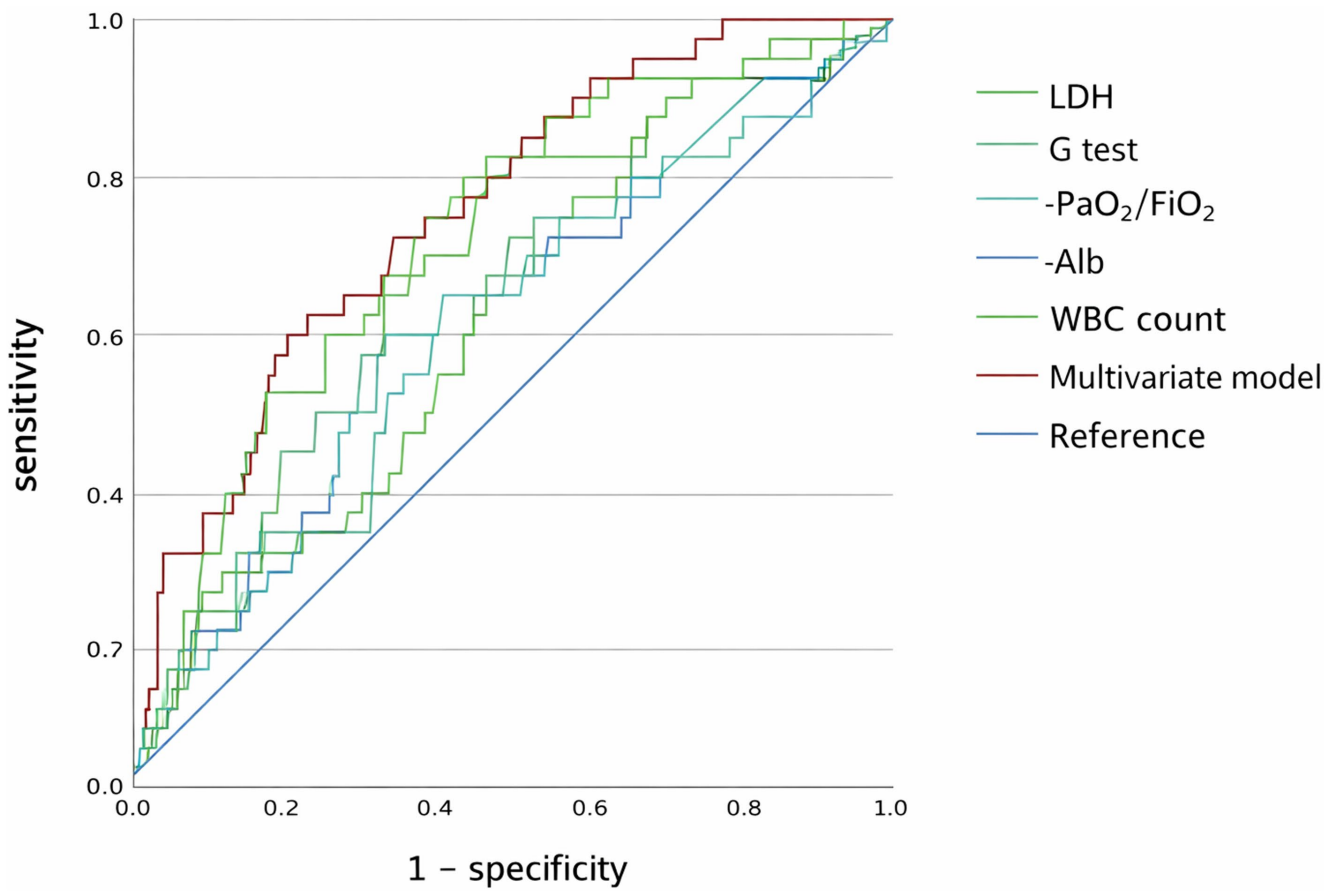
Receiver operating characteristic (ROC) curve analysis of individual indicators (WBC count, LDH, *G* test, PaO_2_/FiO_2_, Alb) and the multivariate model for predicting barotrauma in non-HIV-infected patients with PJP. The *x*-axis denotes 1−specificity (false-positive rate), and the *y*-axis denotes sensitivity (true-positive rate). The blue diagonal line represents the reference line for random prediction. WBC, white blood cell; LDH, lactate dehydrogenase; *G* test, 1,3-beta-D-glucan assay; Alb, albumin.

As shown in Table 6, the final model included the following covariates: sex, age, smoking history, WBC count, LDH, Alb, PaO_2_/FiO_2_, and standardized *G* test levels. Multivariable analysis revealed that a lower PaO_2_/FiO_2_ was significantly and independently associated with barotrauma development. The predictive model derived from this analysis showed moderate discriminative capacity, with an area under the curve (AUC) of 0.751 (95% CI 0.674–0.828) (Figure 2).

**Table 6:** Multivariate logistic regression of independent factors associated with barotrauma in non-HIV PJP patients.

Variables	Multivariate logistic regression		
	OR	95% CI	*P*
Sex	0.828	0.316–2.168	0.700
Age	0.988	0.965–1.012	0.339
Smoking history	2.328	0.911–5.954	0.078
WBC count	1.065	0.970–1.170	0.186
LDH	1.001	1.000–1.002	0.147
*G* test	1.464	0.944–2.270	0.088
Alb	1.001	0.945–1.060	0.974
PaO_2_/FiO_2_	0.993	0.989–0.998	0.003

WBC, white blood cell; LDH lactate dehydrogenase; *G* test, 1,3-β-D-glucan test; Alb, albumin; PaO_2_/FiO_2_, oxygenation index.

## 4 Discussion

Pulmonary barotrauma is a potentially life-threatening complication in PJP patients, often resulting in increased mortality, prolonged hospital stay, and the need for invasive interventions such as chest drainage or mechanical ventilation (4, 5, 7, 10, 11). In our cohort, the overall incidence of pulmonary barotrauma among non-HIV PJP patients was 12.0%, and patients who developed barotrauma had significantly worse clinical outcomes, demonstrating higher rates of ICU admission and in-hospital mortality. On multivariable analysis, a lower PaO_2_/FiO_2_ emerged as an independent factor significantly associated with barotrauma occurrence. The resulting predictive model demonstrated moderate discriminative ability, with an AUC of 0.751 (95% CI 0.674–0.828). While prior research has established the relevance of barotrauma in PJP populations, evidence regarding its incidence, predictors, and clinical implications in non-HIV patients remains limited. A small-scale retrospective study (*n* = 119) in a non-HIV cohort highlighted barotrauma as an emerging concern but failed to establish robust risk factors or predictive tools (7). Our study addresses this gap by utilizing the largest dataset to date focused on this complication in non-HIV PJP patients and by developing a predictive model based on readily accessible clinical parameters (12, 13).

In our study, non-HIV PJP patients who developed pulmonary barotrauma exhibited higher LDH levels, elevated *G* test values, alongside poorer oxygenation (lower PaO_2_/FiO_2_). These findings are partially consistent with previous literature. A meta-analysis by Wang et al. (5) confirmed that elevated LDH, concurrent pulmonary disease, mechanical ventilation, and pneumothorax—were significantly associated with poor prognosis. These studies highlight that lung structural injury and severe disease status collectively contribute to the development of barotrauma and its prognostic implications, which is highly consistent with our findings. However, mounting evidence suggests that the underlying pathogenesis of pulmonary barotrauma extends beyond ventilatory pressure alone and may chiefly involve structural fragility and inflammation-induced alveolar rupture. Elhakim et al. (14) highlighted in a case report that severe diffuse alveolar damage can precipitate pulmonary interstitial emphysema and spontaneous pneumomediastinum through the Macklin effect, even without any mechanical ventilation intervention. Similarly, Huang et al. (15) described a non-HIV PJP patient who developed spontaneous pneumomediastinum, subcutaneous emphysema, and pneumothorax prior to the initiation of mechanical ventilation, implicating intrinsic alveolar disruption rather than ventilator-induced barotrauma. In a larger cohort study, Chung et al. (16) reported that in non-HIV PJP patients requiring ICU care, the incidence of pneumothorax was unrelated to ventilator settings but correlated with CMV co-infection and pre-existing pulmonary pathology. Taken together, these observations bolster the hypothesis that disease severity and alveolar structural integrity—rather than ventilatory pressures per se—play a pivotal role in barotrauma development in PJP. Our study confirms that it also occurs in non-HIV PJP patients managed with conventional oxygen therapy; this observation thereby provides support for this hypothesis. In addition, multivariable analysis revealed a significant independent association between a lower PaO_2_/FiO_2_ at admission and the development of barotrauma. This finding underscores the role of baseline gas exchange impairment as a surrogate for disease severity and alveolar fragility.

Compared to studies in HIV-infected cohorts, the barotrauma rate in our non-HIV population was significantly higher, possibly due to the more acute disease course, greater inflammatory response, and delayed diagnosis often observed in non-HIV PJP (17). Previous studies have shown that non-HIV PJP patients are more likely to present with respiratory failure and require ICU admission, factors that are themselves associated with elevated barotrauma risk (18, 19).

The most important strength of our results is to recognize the risk of barotrauma in patients with PJP by simple and readily accessible clinical indicators, such as PaO_2_/FiO_2_. It carries several important implications. First, early identification of high-risk patients justifies the need for enhanced monitoring and prompt radiographic evaluation early during mechanical ventilation, given that 33.3% of pneumothoraces in our cohort occurred within 72 h of IMV and 23.8% within 24 h of NIV. For instance, routine bedside chest radiography or lung ultrasound may be justified in this subset, especially during periods of respiratory status fluctuation. Furthermore, respiratory support strategies must be carefully individualized. In high-risk patients with a lower PaO_2_/FiO_2_ at admission, clinicians should consider the early implementation of lung-protective ventilation strategies, including tidal volume and positive end-expiratory pressure limitation. Although NIV is often perceived as lower risk, our findings indicate that barotrauma can occur even under non-invasive support, highlighting the need for caution across all forms of ventilatory support. Additionally, these findings can inform prognostic discussions with patients and their families. Given that the in-hospital mortality rate among patients who developed barotrauma in our cohort exceeded 70%, the early identification of at-risk individuals may facilitate shared decision-making and goal-concordant care.

This study has several limitations inherent to its retrospective, single-center design. The potential for selection bias may limit the generalizability of our findings. Although we implemented a rigorous chart review process with dual-physician adjudication, residual confounding from unmeasured variables may persist. Furthermore, our analysis focused on admission variables and did not account for dynamic clinical trajectories, including evolving oxygen requirements, adjustments in ventilator parameters, or radiographic progression. Additionally, the absence of baseline pulmonary imaging or spirometry data represents a limitation, as such information could have helped clarify the role of pre-existing lung conditions in barotrauma risk. Finally, although the prediction model demonstrated moderate discriminative ability, external validation in independent, multicenter cohorts is warranted before clinical implementation.

## 5 Conclusion

This study establishes that pulmonary barotrauma is a common and clinically significant complication in non-HIV PJP. By identifying key risk factors and developing a predictive model, we provide novel insights into pathophysiology and potential avenues for early intervention.

## Data Availability

The data analyzed in this study is subject to the following licenses/restrictions: the datasets generated and analyzed during the current study contain confidential clinical information and are therefore not publicly available. Data can be obtained from the corresponding author upon reasonable request, subject to institutional review board approval and compliance with patient privacy and data protection regulations. Requests to access these datasets should be directed to JM, majjmail@163.com.
